# Arterial stiffness in adult patients after Fontan procedure

**DOI:** 10.1186/1476-7120-12-15

**Published:** 2014-04-10

**Authors:** Lidia Tomkiewicz-Pajak, Hanna Dziedzic-Oleksy, Jacek Pajak, Maria Olszowska, Jacek Kolcz, Monika Komar, Piotr Podolec

**Affiliations:** 1 Institute of Cardiology, Jagiellonian University Medical College and John Paul II Hospital, 80 Pradnicka St., 31-202 Krakow, Poland; 2 Department of Pediatric Cardiology, Silesian Pediatric Medical Center, Katowice, Poland; 3 Department of Pediatric Cardiac Surgery, Polish-American Children’s Hospital, Krakow, Poland

**Keywords:** Adult, Fontan patients, Arterial stiffness

## Abstract

**Objectives:**

Increased arterial stiffness is a risk factor of atherosclerosis and cardio-vascular complications. The aim of the study was to determine whether peripheral vascular function might be an early marker of impaired health status in patients with a single ventricle after Fontan procedure.

**Methods and results:**

Twenty five consecutive adults (11 women and 14 men) aged 24.7 ± 6.2 years after the Fontan procedure and 25 sex, age and BMI match healthy volunteers underwent physical examination, blood analysis, transthoracic echocardiography and noninvasive assessment of aortic stiffness. Augmented pressure and Augmentation Index (AIx) were both significantly elevated in Fontan when compared to the controls (6,08 ± 0,7 vs. 2,0 ± 3,7; p = 0.002 and 17,01 ± 3,3 vs. 6,05 ± 11; p < 0.001, respectively). There were no differences in pulse wave velocity (PWV), mean blood pressure (BP), brachial pulse pressure (PP), central: systolic BP, diastolic BP and PP. In Fontan group we find negative correlation between PWV and SatO2 (r = −0.68; p = 0.04) and positive correlation with WBC (0.72; p = 0.72; p = 0.013), INR (0.81; p = 0.008), TNFα (r = 0.45; p = 0.04), and postoperative time (r = 0.77; p = 0.02). AIx correlates positively only with age at surgery (r = 0.45; p = 0.04). Bilirubin level correlates positively with brachial PP (r = 0.71; p = 0.02) and central PP (r = 0.68; p = 0.03).

The multivariate model showed that SatO2 (β = −0.44, p = 0.04) was the only independent predictor of PWV (R^2^ = 0.32, p = 0.03).

**Conclusion:**

Adult Fontan patients have an increased arterial stiffness assessed by a noninvasive technique. Low arterial oxygen saturation postoperative time, age at surgery, white blood cells, TNFα and bilirubin level are associated with arterial stiffening in these patients. The combination of blood parameters of the hepatic function and noninvasive measurements of arterial stiffness could be helpful in comprehensive care of patients with Fontan circulation.

## Introduction

Non-invasive measurement of vascular parameters is increasingly used to assess the risk of cardiovascular disease. It is considered that the loss of elasticity of the walls of the arteries, especially the aorta is a marker of the early changes that may lead to the development of atherosclerosis and its following complications (eg, hypertension, stroke, myocardial infarction) in healthy individuals [1]. Increase in arterial stiffness was proven to be caused by several factors such as age, hypertension, hypercholesterolemia, diabetes, and smoking [2-5]. In the detection of early growth of the aortic wall stiffness pulse wave velocity (PWV) and/or augmentation index (AIx) are commonly used [6].

Fontan operation is a widely used method of treatment of patients with single ventricle. The main goal of this procedure is the separation of the pulmonary and systemic circulation. The treatment is multistage and it results in the absence of subpulmonary ventricle. The blood flow through the lungs is effected due to the pressure gradient between the systemic veins and the left atrium and has non-pulsatile character [7,8].

A vast majority of the patients born with a functional single ventricle, who underwent Fontan surgery in early childhood, survive over 20 years [9]. The longer the time since the surgery, the more frequent remote complications are noted [8,9]. Thus patients are at high risk of mortality and morbidity. The development of impaired functional health status is caused by reduced cardiac output, increased systemic vascular resistance, ventricular dysfunction, arrhythmia, and heart failure [7-10].

The present study was designed to evaluate the usefulness of the noninvasive measurement of arterial stiffness to determine whether peripheral vascular function might be an early marker of impaired health status in patients with a single ventricle after Fontan procedure. We also evaluated the impact of endothelial dysfunction and inflammation on parameters of arterial stiffness.

## Material and methods

### Study population

The patients were recruited consecutively at the John Paul II Hospital in Krakow and enrolled into the study if they had had the Fontan operation, were ≥18 years old, and were clinically stable for at least 3 months before the study.

The patients underwent physical examination, blood analysis, transthoracic echocardiography and noninvasive assessment of aortic stiffness. Medical histories were taken and they included demographic, anatomic data, previous interventions and medical therapy. The exclusion criteria were: arrhythmia, any acute illness, cancer, diabetes mellitus, hypertension, acute vascular event, alcohol abuse and pregnancy.

Healthy subjects were not on any medication and had no history of cardiovascular or cerebrovascular disease.

The institutional ethics committee approved the study protocol. All the participants signed informed consent before enrolling into the study.

### Arterial stiffness

Assessment of the central hemodynamic data and aortic stiffness was performed by non-invasive examination of the peripheral arteries. Measurements were performed after 8 hours fasting, before medications, in the supine position, in a quiet room, with an air temperature of 22 ± 1°C [6]. Shortly before the test arterial blood pressure (BP) and heart rate were measured with use of an automatic sphygmomanometer OMRON M6. Tests were performed by applanation tonometry by SphygmoCor ® (ATCOR Medical, Sidney, Australia). In order to measure PWV the applanation tonometer was applied at first to the common carotid artery, then to the femoral. The measurements were performed independently of each other but with respect electrocardiography (ECG) registered at the same time.

Central: systolic BP, diastolic BP, pulse pressure (PP), augmented pressure (AP) and augmetation Index (AIx) were obtained during the course of at least 12 seconds registration of the pulse wave graph at the radial artery and calculated by a validated generalized transfer function.

**Figure 1:**
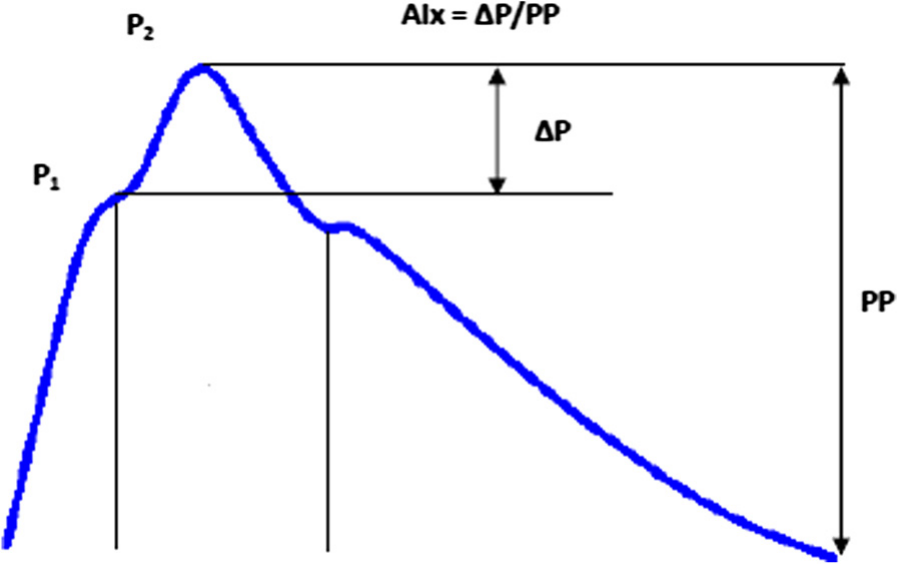
**The pulse waveform in the ascending aorta.** P1 - the first systolic peak caused by the force of myocardial contraction, P2 - the second peak caused by pulse wave reflected returning from the periphery to the heart, AP (AP = P1 - P2) - augmented pressure, PP - (pulse pressure).

AP is a parameter that determines the difference between the peak of the reflected wave from the periphery (P2) and the peak of the wave generated by the contraction of the left ventricle (P1) (AP = P2 - P1 [mmHg]). AP describes the increase or decrease in the amount of pulse wave in the ascending aorta in relation to strengthening it by the wave returning from the periphery to the ascending aorta (Figure 1).

AIx is expressed as the ratio of the augmented pressure (AP) and PP in the aorta. (AIx = AP/PP [%]), and speaks of the percentage change (increase or decrease) of the pressure in the aorta (pulse wave height) under the influence of the peripheral wave and it is dependent on the duration of the cardiac cycle, PWV in the vessels and the amplitude of the reflected wave. Because the value of AIx is influenced by heart rate, AIx was standardized for the heart rate of 75/min [11].

Single ventricle ejection fraction (SVEF) was assessed by echocardiography (Vivid 7 GE Medical System, USA) using a Simpson method. Oxygen saturation (SatO2) was measured noninvasively by pulse oximeter in room air

### Laboratory measurements

Blood samples from a peripheral vein were drawn into tubes. All blood samples were obtained in the morning after an overnight fast. In patients using warfin, blood was drawn at least 5 days after drug withdrawal. Plasma levels of white blood cells (WBC), platelet count, red blood cells (RBC), hematocrit (HCT), hemoglobin (Hg), total protein, alanine aminotransferase (ALT), C-reactive protein (CRP), creatinine, bilirubin, gamma glutamylo transpeptidaza (GGTP) international normalized ratio (INR), N-terminal pro-B-type natriuretic peptide (NT-pro-BNP) were assayed by routine laboratory techniques.

Endothelin-1 level and tumor necrosis factor-alfa (TNFα) were determined by the ELISA method (R&D Systems).

### Statistical analysis

Continuous variables are expressed as mean ± SD. Categorical variables are described as counts and percentages. Patients after Fontan operation and controls were compared with the Mann Whitney U-test for the continuous variables and with the chi square test for categorical variables. Correlations between the individual parameters were calculated using the Spearman rank test. Factors that determine PWV were analyzed using multiple logistic regression. A p-value <0.05 was considered statistically significant. The statistical analyses were performed with the PQ Stat version 1.4.2.324 software.

## Results

### Group characteristics

Of 35 patients who were assessed for eligibility, 10 subjects were excluded due to: age below 18 years of life (n = 1), lack of informed consent (n = 3), and arrhythmias (n = 6). The study population consisted of: 25 consecutive adults (11 women and 14 men) aged 24.7 ± 6.2 years who underwent the Fontan procedure in their childhood and 25 sex, age and BMI match healthy volunteers. The dominant cardiac malformation were: tricuspid atresia 12 (48%), pulmonary atresia with ventricular septal defect and transposition of great arteries 8 (32% ), double outlet right ventricle with hypoplasia of left ventricle 2 (8%), double inlet left ventricle 3 (12%). Modification of the Fontan operation included direct right atrium-pulmonary artery connection in 2 (8%) patients and total cavopulmonary connection (TCPC) by means of intraatrial lateral tunnel in the remaining 23 (92%) patients. The mean age at Fontan procedure was 4.6 ± 3.3 years. The mean follow-up time was 20.1 ± 5.2 years. The patients were in NYHA class I 5 (20%), NYHA II 18 (72%) and NYHA III 2 (8%). Six (24%) patients were treated with warfarin, 1 (4%) with enoksaparin, 15 (60%) with aspirin and 3 (12%) were taking inhibitor of angiotensin in a small doses. On the day of examination the patients did not take any medication.

The Fontan patients had lower SVEF (51.6 ± 5.4 vs. 69.3 ± 1.6%; p < 0.001, SatO2 (88.5 ± 6.8 vs. 96.9 ± 1.6%, p < 0.001) and there were no differences in SBP, DBP, HR. Study population characteristic is shown in Table 1.

**Table 1:** Study population characteristic

	**Fontan (n = 25)**	**Healthy (n = 25)**	**p=**
Age, years	24.7 ± 6.2	26.9 ± 3.4	0.14
Sex	14 M / 11 F	15 M / 10 F	0.4
BMI, kg/m^2^	21,7 ± 2	22,56 ± 3	0.3
SBP, mmHg	127.2 ± 13.9	124 ± 10.6	0.42
DBP, mmHg	79.4 ± 7.3	77.7 ± 5.2	0.32
HR, BPM	64.8 ± 12.6	68.9 ± 16.2	0.33
SatO2,%	88.5 ± 6.8	96.9 ± 1.6	<0.001
SVEF,%	51.6 ± 5.4	69.3 ± 1.6	<0.001

BMI-body mass index, SBP-systolic blood pressure, DBP- diastolic blood pressure, HR- heart rate, SatO2 – arterial saturation, SVEF – single ventricle ejection fraction.

In Fontan group we also find significant higher RBC (6.2 ± 2.5 vs. 4.8 ± 0.5 10^9^/μl; p < 0.001), Hg (16.8 ± 1.4 vs. 14.1 ± 1.3 g/dl; p < 0.001), HCT (49.1 ± 3.3 vs. 42.2 ± 3.3%; p < 0.001), GGTP (87.3 ± 50.3 vs. 17.8 ± 8.3%; p < 0.001), ALT (30.9 ± 11.9 vs. 21.3 ± 6.2 IU/l; p < 0.001), bilirubin (23.7 ± 16.3 vs. 8.1 ± 3.8 μmol/l; p < 0.001), INR (1.2 ± 0.2 vs. 1 ± 0.1; p < 0.001), TNFα (7.1 ± 1.5 vs. 2.4 ± 1.2 pg/ml; p < 0.001), NT-proBNP (302.3 ± 601.8 vs. 72.2 ± 45.9 pg/ml; p < 0.001), endothelin levels (2.5 ± 0.1 vs. 1.3 ± 0.05 pg/ml; p = 0.002) and significantly lower platelet account (127.8 ± 33.8 vs. 228.1 ± 50.6 10^9^/μl; p = <0.001). There were no differences in WBC, total protein, creatynin and CRP. The results are shown in Table 2.

**Table 2:** Laboratory characteristics of Fontan patients and controls

**Variable**	**Fontan (n = 25)**	**Controls (n = 25)**	**p**
WBC, 10^3^/μl	5.2 ± 1.6	5.9 ± 1.4	0.1
RBC, 10^9^/μl	6.2 ± 2.5	4.8 ± 0.5	<0.001
Hg, g/dl	16.8 ± 1.4	14.1 ± 1.3	<0.001
HCT,%	49.1 ± 3.3	42.2 ± 3.3	p < 0.001
Platelet count, 10^3^/μl	127.8 ± 33.8	228.1 ± 50.6	<0.001
Total protein, g/dl	75.9 ± 4.9	73.9 ± 2.5	0.08
GGTP, U/L	87.3 ± 50.5	17.8 ± 8.3	<0.001
ALT, IU/l	30.9 ± 11.9	21.3 ± 6.2	<0.001
Bilirubin, μmol/l	23.7 ± 16.3	8.1 ± 3.8	<0.001
INR,	1.2 ± 0.2	1 ± 0.1	<0.001
TNFα, pg/ml	7.1 ± 1.5	2.4 ± 1.2	<0.001
CRP, mg/l	2.6 ± 2.7	1.9 ± 1.7	0.6
Endthelin-1, pg/ml	2.5 ± 0.1	1.3 ± 0.05	0.002
NTpro-BNP	302.3 ± 601.8	72.2 ± 45.9	<0.001

WBC- white blood cells, RBC - red blood cells, Hg – hemoglobin, HCT- hematocrit, GGTP – gammaglutamylo transpeptydaza, ALT - alanine aminotransferase, INR - international normalized ratio, TNFα – tumor necrosis factor alfa, CRP - C-reactive protein, NT-pro-BNP- N-terminal pro-B-type natriuretic peptide.

### Arterial stiffness

Augmented pressure and Augmentation Index were both significantly elevated in Fontan when compared to the controls (6.08 ± 0.7 vs. 2.0 ± 3.7; p = 0.002 and 17.01 ± 3.3 vs. 6.05 ± 11; p < 0.001, respectively). There were no differences in PWV, Mean BP, brachial PP, Central SBP, Central DBP, Central PP. The results are presented in Table 3.

**Table 3:** Arterial stiffness measurements

	**Fontan (n = 25)**	**Healthy (n = 25)**	**p=**
PWV (m/s)	7,81 ± 1,0	7,91 ± 0,8	0.34
AP	6,08 ± 0,7	2,0 ± 3,7	0.002
AIx	17,01 ± 3,3	6,05 ± 11	<0.001
Mean BP, mm Hg	94,5 ± 8,61	92,2 ± 6,9	0,29
PP brachial, mm Hg	47,4 ± 13,8	46,2 ± 11,5	0,74
Central SBP, mm Hg	113,4 ± 12,3	107,9 ± 9,0	0,07
Central DBP, mm Hg	80.4 ± 7,6	78,1 ± 5,4	0,21
Central PP, mm Hg	33,0 ± 10,5	29,8 ± 6,7	0,19

PVW- Pulse Wave Velocity, AP- Augmented Pressure, AIx- Augmentation Index, BP – blood pressure, PP- pulse pressure, SBP – systolic blood pressure, DBP- diastolic blood pressure.

In Fontan group we find negative correlation between PWV and SatO2 (r = −0.68; p = 0.04) and positive correlation with WBC (r = 0.72; p = 0.72; p = 0.013), INR (r = 0.81; p = 0.008), TNFα (r = 0.45; p = 0.04), and postoperative time (r = 0.77; p = 0.02). We did not find similar correlation in controls.

Augmentation Index correlates positively only with age of surgery (r = 0.45; p = 0.04). Bilirubin level correlates positively with PP brachial (r = 0.71; p = 0.02) and Central PP (r = 0.68; p = 0.03).

To determine the independent effect of clinical and laboratory variables, the stepwise regression analysis for PWV as a dependent variable was performed. The multivariate model showed that SatO2 (β = −0.44, p = 0.04) was the only independent predictor of PWV (R^2^ = 0.32, p = 0.03).

## Discussion

In the present study of arterial stiffness in patients after Fontan procedure, we showed increased Augmentation Index and no difference in Pulse Wave Velocity between Fontan patients and healthy population. In a healthy cardiovascular system, the flexible wall of the aorta increases its volume during the systolic ejection of blood from the left ventricle. This feature prevents excessive increase in systolic blood pressure. Then, in the diastole aortic volume it gradually returns to the original size, ensuring the free flow of blood towards the peripheral vessels. Pulse wave, created in this way, reaches the distal parts of the circulatory system, and reflects back towards the heart, reaching the ascending aorta during the next diastole phase. Its return strengthens the new pulse wave, causes the increase in diastolic blood pressure, and provides adequate perfusion of the coronary arteries [6]. The aorta acts as a conduit and a cushion for pulsate flow of the blood, and any stiffening of its wall results in faster propagation of the blood (i.e., increased PWV). Arterial stiffness represents the load against which the left ventricle must eject its volume.

Arterial stiffness may be evaluated in any part of the arterial system. However, the process of stiffening that occurs in the walls of the aorta is the one of the primary interest [6]. In literature there is evidence that increased arterial stiffness is an independent predictor of morbidity and mortality in patients with cardiovascular diseases as well as in healthy individuals [12-16].

The idea that the biophysical properties of the aorta are abnormal in a population of patients with Fontan circulation has been recently studied by Myers at al [17]. Their research showed that Fontan patients had faster PWV, implying stiffer arteries and abnormal arterial health. They concluded that increased arterial stiffness is an additional factor responsible for the development of late ventricular failure in patients with single ventricles.

Our data are similar to the results of Lambert et al. [18]. The authors not only showed the increased arterial stiffness comparing to healthy controls, but also demonstrated that Fontan patients have increased muscle sympathetic nerve activity and impaired endothelial function too. They postulated that monitoring the development of the increased arterial stiffness along with clinical outcomes may help in delay of the inevitable diastolic dysfunction of the Fontan patients’ heart.

The mechanism leading to an increased arterial stiffness in Fontan patients is difficult to define. It was proven that parameters of the arterial stiffness are under the influence of age [17]. The results of our study do not confirm the relationship between abnormal arterial stiffness and patients age. Moreover, we find association between parameters of arterial stiffness and postoperative time in patients with Fontan circulation. There is evidence that majority of complications appear late after Fontan procedure [8,10]. We also find association between augmentation index and age at surgery. The results are not surprising, given the fact that before the surgery, chronic cyanosis and volume overloading may contribute to arterial stiffness [7].

Our study demonstrated that arterial oxygen saturation is an important determinant of arterial stiffness in adult Fontan patients. To our knowledge there was no research in this area. However, the problem was discussed in literature concerning patients with sleep-disordered breathing as well as in patients with chronic obstructive pulmonary disease (COPD). The authors [19,20] found that nocturnal O2 desaturation caused by sleep apnea is associated with increased PWV among normotensive and hypertensive individuals. Cinarka et al. [21] not only showed that hypoxemia affects arterial stiffness, but also proved that PWV is faster in patients with severe COPD than in patients with milder forms of this disease.

Previous study showed that arterial oxygen saturation decreased in majority of adult Fontan patients [8]. The reason of this phenomena is multifactorial and may result from a progressive impairment of single ventricle function, a rise in pulmonary pressure and right-to-left shunt through an atrial fenestration or venovenous collaterals [8,9]. In our study fenestration was an open in 25% of patients. Wykretowicz et al [22] showed that in adult patients with cyanotic congenital heart diseases arterial stiffness is affected by hematocrit concentration but not SatO2. However, our study did not find such correlation It should be noted that presented patients with cyanotic congenital heart diseases had higher mean hematocrit level (64%) than our Fontan patients.

Another factor that may affect the biophysical properties of the aorta and therefore influence the wave reflection, arterial pressure augmentation and contribute to central and peripheral vascular dysfunction, is endothelial dysfunction. We find elevated endothelial level in Fontan patients. Jin et al. [23] has shown that the endothelial dysfunction is more prevalent in Fontan patients compared with healthy controls. Also the study of Natarajan et al. [24] which evaluated vascular function in patients with pre-Fontan single-ventricle physiology reported an increased endothelin-1 level and decreased flow-mediated dilation.

Our study showed that also inflammation influenced arterial stiffness in adult Fontan patients. We find correlation between PWV and white blood cells and TNFα. There is limited data regarding the relationship of inflammation with arterial stiffness in patients with single ventricle. However, previous study demonstrated that increase in systemic inflammation is an independent predictor of future development of hypertension [25]. The increased vascular inflammation may increase vascular fibrosis, cells proliferation, impaired endothelial which lead to increased arterial stiffness [26]. The white blood count is a simple and common test that was also proven to be associated with stiffening of the arterial walls [27]. However all of the studies concerned population with cardiovascular problems such as hypertension, diabetes or coronary artery disease. It has been suggested that an elevated white blood count, even within normal range, is significantly associated with all cause, cardiovascular, and cancer mortality [28]. We believe that this simple test may be useful to determine the entirety condition of the patient with Fontan circulation.

Liver is the organ at great risk after the Fontan operation [29]. Hepatic dysfunction caused by volume overload and low cardiac output may result from a coexistence of passive venous congestion of the liver, hypoxia, or concomitant pulmonary disease [30]. Noninvasive measures of hepatic function include inter alia the measurements of bilirubin, albumin, international normalized ratio. Most patients with congenital heart disease and passive venous congestion of the liver have elevated indirect bilirubin and prolonged international normalized ratio with minimal elevations of the aminotransferases [31]. We have observed the same results in our patients. Moreover, we have demonstrated that these parameters remain in correlation with noninvasive measurement of arterial stiffness. The association of liver disease and increased arterial stiffness and therefore elevated cardiovascular risk has been previously shown in nondiabetic, nonhypertensive individuals [32]. It has been postulated that screening tests that could identify earlier stages of hepatic dysfunction development would be of a great value, especially since currently used tests are relatively insensitive [30]. The combination of blood parameters of the hepatic function and noninvasive measurements of arterial stiffness could be helpful in comprehensive care of patients with Fontan circulation.

### Study limitation

Several limitations of this study should be acknowledged. The study group was small and heterogeneous with regard to type of surgery. Also medication administrated to the Fontan patients is not without an effect on the arterial stiffness. The patients in our study were taking medication such as ACE inhibitors - the drugs that decrease AIx [21]. This may suggest that the actual impact of increased arterial stiffness may be underestimated. However the patients did not take any medication on the day of examination. Moreover, we did not determined single ventricle ejection fraction on magnetic resonance. However, previous studies. demonstrated similar interobserver reproducibility for echocardiographic and cardiac magnetic resonance assesment of single ventricle function. Overall, the cardiac magnetic resonance data were inadequate or incoplite in 30% of patients in whom the test was performer, predominantly, due to metallic artefacts [8,33,34].

## Conclusion

Adult Fontan patients have an increased arterial stiffness assessed by noninvasive technique. Low arterial oxygen saturation postoperative time, age at surgery and bilirubin level are associated with arterial stiffening in these patients. The combination of blood parameters of the hepatic function and noninvasive measurements of arterial stiffness could be helpful in comprehensive care of patients with Fontan circulation.

## Competing interests

The authors declare that they have no competing interests.

## Authors’ contributions

LTP designed the study, collected the data, interpreted the data and drafted the article. HDO participated in the design of the study, collected data and helped to draft the manuscript. JP collected the data and interpreted the data. MO interpreted the data and helped to draft the manuscript. JK collected the data, interpreter the data, MK collected data. PP interpreted the data, helped to draft the manuscript and designed the study. All authors read and approved the final manuscript.
